# Development and validation of oral chemotherapy self-management scale

**DOI:** 10.1186/s12885-020-07404-0

**Published:** 2020-09-16

**Authors:** Qi Peng, Wanying Wu

**Affiliations:** Cancer Hospital of the University of Chinese Academy of Sciences (Zhejiang Cancer Hospital), Institute of Cancer and Basic Medicine (IBMC), Chinese Academy of Sciences, East Banshan Road, Gongshu District, Hangzhou Zhejiang, People’s Republic of China 310022

**Keywords:** Cancer, Oral chemotherapy, Self-management, Validation

## Abstract

**Background:**

With the increase of oral chemotherapy drugs, patients receiving cancer treatment prefer oral chemotherapy versus intravenous, given equal efficacy and toxicity. However, they need to take an active part in their care, which is vital with home-based oral therapy, therefore the self-management is important for patients with oral chemotherapy. Unfortunately, the development of self-management assessment tools for oral chemotherapy still lags behind.

**Methods:**

The OCSMS item pool was formulated based on literature review and semi-structured interviews, An initial scale containing 5 dimensions and 38 items was constructed through research seminar, Delphi survey and pilot testing. To assess the validity and reliability, We recruited 261 patients from cancer hospital in China.

**Results:**

A 36-item scale was developed with five dimensions identified through factor analysis: daily life management, symptom management, medication management, emotional cognitive management and social support. Cronbach’s coefficient Alpha, split-half coefficient, test-retest reliability and S-CVI/UA scores were 0.929, 0.773, 0.966 and 0.833, respectively, indicating that OCSMS has good reliability and validity.

**Conclusions:**

The OCSMS is a valid, reliable measurement method of the self-management ability of patients with oral chemotherapy. The OCSMS shows potential as a tool to ensure the safety of patients with cancer. The OCSMS may help evaluate the effectiveness of interventions to improve the self-management ability of patients.

## Background

Chemotherapy is one of the most important treatments for cancer, and the route of chemotherapy administration is developing continuously. With the spread of oral chemotherapy agents over the last 15 years, people are choosing oral chemotherapy because it is safe, economical and helps prevent venepuncture [1–3]. Patients are likely to choose oral chemotherapy than intravenous chemotherapy even with their same efficacy and toxicity [4].

With oral anticancer agents becoming widely common, a critical shift has occurred from clinic-based healthcare provider-administered management to home-based self-administered management [5]. However, it also brings new challenges. For example, patients at home can’t recognize the undesirable side effects, such as nausea and vomiting [4, 6]. Hence, the patient’s self-management ability is very important.

Self-management was proposed by Corbin and Straus for chronic disease [7] and has been defined as managing symptoms, treatments, lifestyle alterations and psychosocial consequences of health conditions [8]. Self-management has been widely used in all aspects of management. Numerous studies have shown that enabling effective self-management of medication in non-malignant chronic diseases (i.e.,Hypertension) results in better disease control and a better quality of life [9, 10]. Self-management is particularly important for patients with oral chemotherapy because it affects their adherence to the treatment, quality of life and safety [11, 12]. To date, no effective evaluation tools have been designed to measure self-management for oral chemotherapy. The purpose of this study is to develop a valid and reliable instrument oral chemotherapy self-management scale (OCSMS).

## Methods

**Fig. 1:**
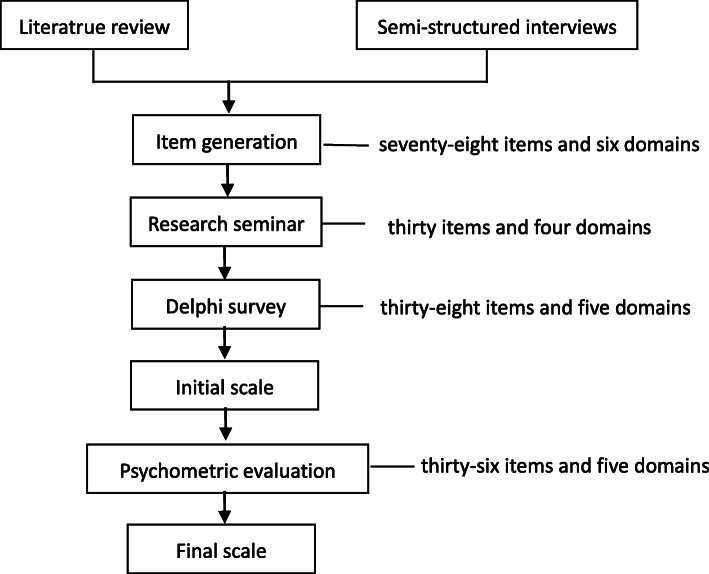
Development of Oral Chemotherapy Self-management Scale

This study is a prospective, mixed-method scale development of oral chemotherapy self-management scale (OCSMS). Figure 1 describes the development of this scale.

### Ethical approval

This study was approved by the medical ethics committee of ZheJiang Cancer Hospital, Hangzhou, Zhejiang, China (IRB-2015-208).

### Item generation

Literature review and semi-structured interviews were conducted to generate an item pool. A comprehensive literature review was performed to generate a semi-structured interview guide, which was used for in-depth interviews [13]. Purpose sampling was adopted, involving 10 experts (including nurses, doctors and pharmacists) and 9 patients. Data were analysed using Nvivo11 software. A total of seventy-eight items and six domains of oral chemotherapy self-management were generated.

### Research seminar

A total of seventy-eight items and six domains were scrutinized during two-round research seminar. Eight experts with a titles of deputy senior or above and bachelor’s degree or above and six experts with master’s degree or above and more than 3 years of relevant work experience were selected in the two-round research. Thirty items and four domains of oral chemotherapy self-management were chosen in the two-round research seminar.

### Delphi survey

Two rounds of Delphi consultation were conducted. Eighteen experts (nurses, doctors and pharmacists) from eight provinces, including Shanghai, Beijing, Zhejiang and Hunan, were invited to evaluate the scale format and each item. All experts have a bachelor’s degree and more than 10 years of relevant work experience. They read, evaluated and rated each item based on its clarity, uniqueness and relevance to oral chemotherapy self-management. Items with a coefficient of variation > 0.25 and average score < 3.5 were removed [14, 15]. We had removed one item, and had added nine items and one domain of oral chemotherapy self-management by using Delphi method.

Additional information on this study design, data analysis and results of literature review and Delphi survey can be found in previous publications [15, 16].

### Pilot testing

Prior to undertaking the psychometric properties of the scale, we recruited 40 participants (20 females) for pilot testing. The mean age of 40 participants from Zhejiang Cancer hospital was 53.05 ± 10.68 years (range 27–73 years). The purpose of the pilot testing is to find possible administration problem, such as miss-phrasing, and to determine which items should be modified, added or removed. The researchers explained the purpose of the study, and gave an example before its completion for each participant. After the patients completed the scale, the researchers asked the patients whether they unable to respond. Through the pilot testing, we modified two items without adding or removing any items.

### Validity and reliability

To evaluate the psychometric properties of the scale, we recruited 261 participants from Zhejiang Cancer hospital in China between May 12,018 and January 312,019. The minimum sample size for factor analysis should be five times the number of items with at least 200 cases [17]. For the test–retest reliability evaluation of the scale, 40 of the participants returned 2–5 weeks later to complete the same survey. Two participants withdrew from the study. The inclusion criteria were as follow: adults (more than 18 years old) with confirmed diagnosis of cancer, using an oral chemotherapy regimen, willing to participate, and able to communicate using Mandarin. The exclusion criterion was inability for self-care.

## Results

### Characteristics of the participants

The mean age of participants was 54.78 years (min = 27, max = 83). Among them, 59.4% were male, and 90.4% were married. Table 1 shows their selected demographic characteristics.

**Table 1:** Demographic Characteristics of the Study Participants

characteristics	N(%) or (Mean ± SD)
Age Mean, years(SD)	54.78 ± 10.03
Gender	
Male	155(59.4%)
Female	106(40.6%)
Civil status	
Married	236(90.4%)
Single	7(2.7%)
Divorced	2(0.8%)
Widowed	9(3.4%)
misses	7(2.7%)
Employment situation	
Unemployed	80(30.7%)
Working	84(32.2%)
Retired	52(19.9%)
others	45(17.2%)
Level of education	
Without studies	26(10.0%)
Primary education	69(26.4%)
Secondary education	135(51.7%)
University education	31(11.9%)
Disease	
Rectum cancer	96(36.8%)
Colon cancer	70(26.8%)
Gastric cancer	47(18.0%)
Breast cancer	20(7.7%)
Others	28(10.7%)
Course of disease, months(SD)	54.84 ± 10.19
Medication	
Capecitabine Capsule	133(51.0%)
Xelode Capsule	66(25.3%)
Tegafur Gimeracil Oteracil Potassium Capsule	55(21.1%)
Others	7(2.7%)

### Validity of the scale

**Fig. 2:**
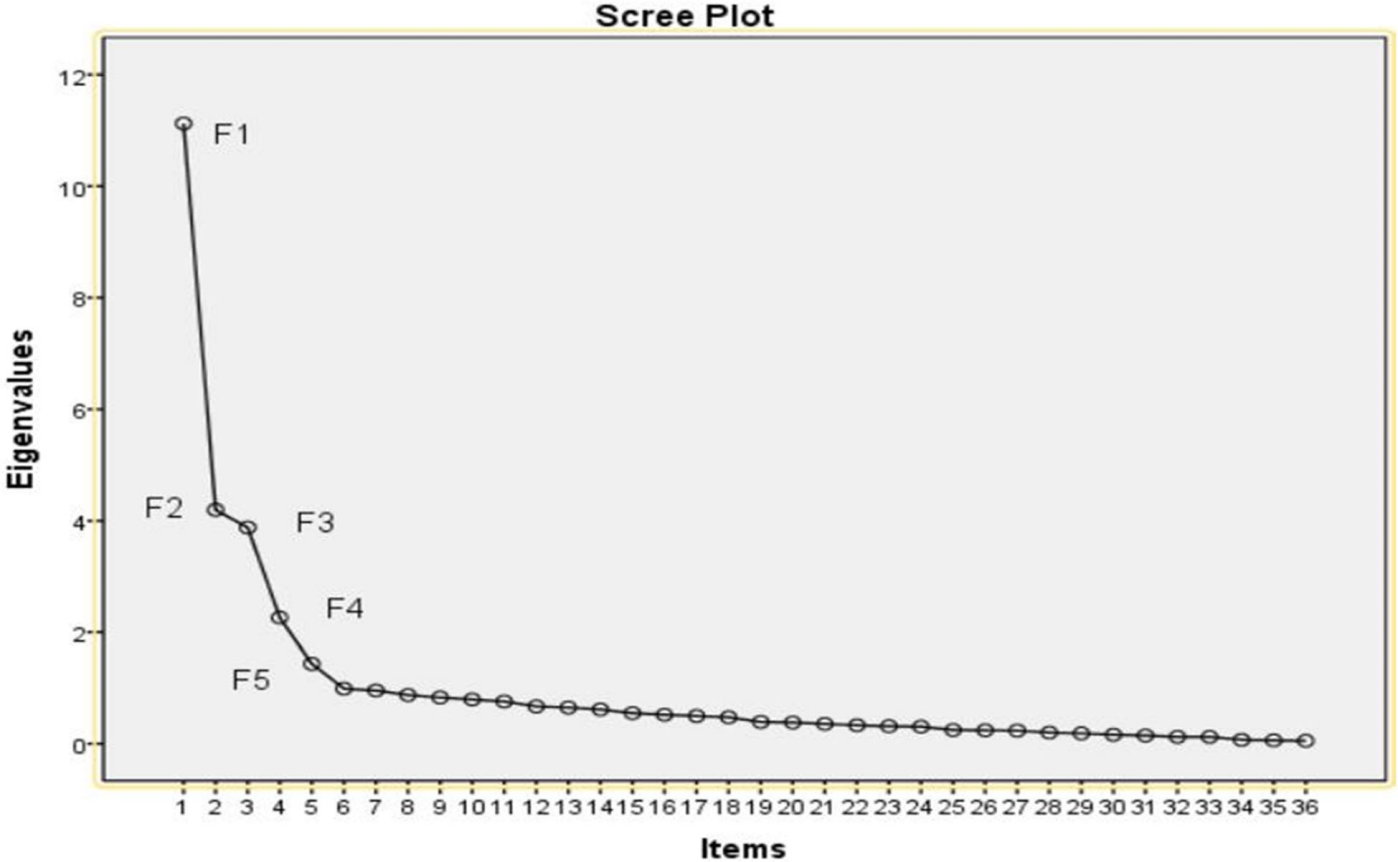
Scree Plot

We removed two items through exploratory factor analysis. The value of KMO was 0.886, and the result of Barlett’s sphericity test was X^2^ = 7106.941 (df = 630, *p* = 0.000). These values indicated that the items of the scale were appropriate for factor analysis. The structure validity of the scale was evaluated with principal components analysis using Varimax rotation. Factors with eigenvalues > 1 were selected. Figure 2 shows that eigenvalues slightly decreased after component 5. Five sub-dimensions were created and labelled as: daily life management (8 items), symptom management (6 items), medication management (11 items), emotional cognitive management (7 items) and social support (4 items). The cumulative explained variance rate of the scale was 63.603%, and the item factor loadings was 0.530–0.903 (Table 2).

**Table 2:** Item Factor Loadings

Domains	Contents	Factor 1	Factor 2	Factor 3	Factor 4	Factor 5
Daily life management	1 pay attention to the diet, choose digestible food high in vitamins (fresh vegetables and fruit) and quality protein (such as fish, meat eggs and milk)	0.648				
	2 frequent small meals, avoid irritating foods (such as spicy, cold food)	0.693				
	3 drink more water; the daily amount of drinking water is more than 2000 ml	0.765				
	4 pay attention to your body weight change every week	0.700				
	5 keep a regular sleep schedule, and guarantee 6–8 h of sleep every day	0.903				
	6 exercise properly based on your own physical condition, such as walking, square dance and Tai Chi	0.783				
	7 your smoking situation during taking oral chemotherapy	0.855				
	8 your drinking situation during taking oral chemotherapy	0.815				
Symptom management	9 understand the common adverse reactions of oral chemotherapy drugs		0.768			
	10 readily identify the adverse symptoms of oral chemotherapy drugs.		0.810			
	11 assess the severity of adverse symptoms caused by oral chemotherapy		0.699			
	12 when a mild adverse reaction occurs, simple measures can be taken.		0.743			
	13 when serious adverse reaction occurs, you can contact doctor timely.		0.606			
	14 follow the doctor’s advice and return to the hospital for regular review.		0.582			
Medication management	15 understand the relevant knowledge of oral chemotherapy drug (including drugs and course)			0.842		
	16 actively consult on the knowledge and requirements of medication at the time of treatment			0.896		
	17 accept doctors’ medication plan			0.814		
	18 other daily information (such as other patients and advertisements) that will affect your choice of medication plan			0.558		
	19 store medication according to drug storage conditions, such as light, moisture and temperature requirements			0.687		
	20 check the completeness of the drug prior to ingestion (such as the completeness of outer packaging and tablet defects)			0.544		
	21 adjust the dosage according to the doctor’s instructions			0.701		
	22 take medicine on time according to the doctor’s advice			0.890		
	23 does not touch the chemotherapy drugs when taking the medicine			0.860		
	24 the excrement can be cleaned up in time, and the toilet is continuously flushed twice during the treatment			0.693		
	25 understand the treatment of the remaining oral chemotherapy			0.612		
Emotional cognitive management	26 able to communicate with friends or colleagues				0.770	
	27 feel that my friends or colleagues treat me differently				0.892	
	28 unconsciously vent my emotions to my family or friends during the treatment				0.764	
	29 does not want to talk to anyone when feeling depressed				0.805	
	30 can relieve stress through talking, watching TV, surfing the Internet, taking a deep breath and meditating				0.779	
	31 learn that negative emotions affect your body				0.833	
	32 able to recognize their own emotional changes				0.577	
Social support	33 be cared for and supported by family, friends or medical personnel					0.530
	34 actively participate in social activities within their capacity.					0.616
	35 actively exchange medication information with family members or caregivers					0.691
	36 actively communicate disease treatment information with medical personnel.					0.688

A four-point (1 = not relevant, 2 = weak relevant, 3 = strong relevant, 4 = very relevant) ordinal rating scale was used. The Scale-level CVI/ universal agreement (S-CVI/UA) and the item-level CVI (I-CVI) of scale were calculated. According to the number of votes that each item received from the panel of 6 experts, S-CVI/UA was 0.833 and I-CVI of scale ranged from 0.833 to 1(Table 3). The value of S-CVI/UA > 0.8 and I-CVI ≧0.78 mean good content validity.

**Table 3:** Experts’ Ratings and CVI Calculation (*N* = 6)

Item	Experts Ratings						Number of 3 or 4 items	I-CVI
	A	B	C	D	E	F		
1	3	4	4	4	4	4	6	1
2	3	4	4	4	4	4	6	1
3	3	4	4	4	4	4	6	1
4	3	3	4	3	4	4	6	1
5	3	3	4	3	4	4	6	1
6	3	3	4	4	4	4	6	1
7	3	3	3	2	3	3	5	0.83
8	2	3	3	3	3	3	5	0.83
9	4	4	4	4	4	4	6	1
10	4	4	4	4	4	4	6	1
11	4	4	3	4	4	4	6	1
12	4	4	4	4	4	4	6	1
13	4	4	4	4	4	4	6	1
14	4	4	4	4	4	4	6	1
15	4	4	4	4	4	4	6	1
16	4	3	3	4	4	4	6	1
17	4	4	3	4	4	4	6	1
18	4	4	4	3	4	4	6	1
19	4	3	4	3	4	4	6	1
20	4	4	4	4	4	4	6	1
21	4	4	4	4	4	4	6	1
22	4	4	4	4	4	4	6	1
23	4	4	4	4	4	4	6	1
24	4	3	4	4	4	4	6	1
25	4	4	4	4	4	4	6	1
26	3	2	3	3	3	3	5	0.83
27	3	3	2	3	3	3	5	0.83
28	3	4	4	4	3	3	6	1
29	3	3	4	4	4	4	6	1
30	3	3	4	4	4	4	6	1
31	3	3	4	4	4	4	6	1
32	3	3	4	3	4	4	6	1
33	3	3	4	3	4	4	6	1
34	3	3	4	3	3	3	6	1
35	4	4	3	3	4	2	5	0.83
36	3	4	3	3	2	3	5	0.83

### Reliability of the scale

The internal consistency coefficient “Cronbach’s Alpha” of total scale was 0.929 with the Cronbach’s Alpha of each factor at 0.664–0.927. The split-half coefficient of the total scale was 0.773 with the split-half coefficient of each factor at 0.584–0.919 (Table 4). The test-retest reliability of the total scale was *r* = 0.966, which was statistically significant and indicated that the scale had good stability over time.

**Table 4:** Cronbach’s Alpha and Split-half Coefficient of each Domain

Domains	The Cronbach’s Alpha	The split-half coefficient
Daily life management	0.920	0.858
Symptom management	0.869	0.865
Medication management	0.927	0.901
Emotional cognitive management	0.904	0.919
Social support	0.664	0.584

## Discussion

### Reliability and validity of the scale

The OCSMS exhibited good reliability and validity in a clinical sample of patients with oral chemotherapy. We calculated the Cronbach’s Alpha to evaluate the unidimensionality of a set of items. The Cronbach’s Alpha of OCSMS was 0.929, suggesting that the items have relatively high internal consistency. The split-half coefficient and test–retest reliability were also high, implying that the OCSMS has good reliability.

The CVI of OCSMS was quite high, indicating that its items adequately represent the construct being measured. Factor analysis of the OCSMS revealed that the five sub-dimensions accounted for 63.603% of the total variance.

### Sub-dimensions of scale

Some scholars believe that oral chemotherapy has the advantages of convenience and tolerance; however, its side effects still need to attract the attention of medical workers [18]. In the interview of this study, patients with cancer receiving oral chemotherapy reported that the most important thing is the lack of professional guidance, especially on adverse symptoms and medications. Therefore, the sub-dimensions of scale include symptom and medication management. Another is that patients have negative emotions, which may be related to the high mortality rate of the tumour. Negative psychology can induced the disease to deteriorate. Therefore, the indicators of this study include emotional cognitive management. On the basis of literature review and expert panel, five sub-dimensions were finally labelled as: daily life management, symptom management, medication management, emotional cognitive management and social support.

### Implications for clinical practices

Oral cancer therapies have several advantages, including great flexibility and convenience for the patient and minimal disruption of daily activities [19]. However, some shortcomings, such as the need to self-manage drugs and identify adverse drug reactions, are also noted. Therefore, the patients’ self-management ability is important. Lack of self-management can lead to adverse consequences that may affect therapeutic outcomes and patients [20]. Existing research focused on patient’s compliance [21–23]. Prior to this study, no instrument has been designed to assess the self-management ability of patients with oral chemotherapy. A qualitative study [24] showed that self- management should be assessed for patients with oral chemotherapy; hence, a tool for this aspect must be developed. Nurses can give proper health education to patients with poor self-management ability. This tool could be used to ensure the safety of patients with cancer receiving oral chemotherapy at home.

### Study limitations

Our study has a number of limitations. First, The research was conducted only in China. Cross-cultural validation studies are necessary. Second, Although the OCSMS was found to have a five-factor structure and good reliability, more research is needed to establish its concurrent or convergent validity or discriminant validity. Third, The sample size (*n* = 261) was 6.87 times the number of items (38 times), so further studies with larger sample size is required to validate the OCSMS.

## Conclusion

The OCSMS has high reliability and validity and takes only a few minutes to complete. Patients with cancer receiving oral chemotherapy reported that this scale is easy to take and can evaluate their self-management ability.

## Data Availability

All data supporting the findings are included in this publication.

## Abbreviations

OCSMS: Oral chemotherapy self-management scale
KMO: Kaiser-meyer-olkin
CVI: Content validity index
S-CVI/UA: Scale-level CVI/ universal agreement
I-CVI: Item-level CVI
